# How can low-carbon help high-quality urban development?—Empirical evidence from low-carbon city pilot policies

**DOI:** 10.1371/journal.pone.0302683

**Published:** 2024-05-06

**Authors:** Dongping Fu, Liuting Zhang

**Affiliations:** School of Economics, Guizhou University of Finance and Economics, Guiyang, Guizhou, China; Laboratoire Ville Mobilite Transport, FRANCE

## Abstract

High-quality development is the paramount task for comprehensively building a socialist modernized country. The low-carbon city pilot policy, with cities as the unit of action, provides new opportunities for high-quality economic transformation. Based on panel data from 261 cities between 2005 and 2018, this study calculates the level of high-quality economic development in Chinese cities, constructs a multi-period difference-in-differences model, analyzes the impact of the low-carbon city pilot policy on high-quality economic development, and explores the policy’s heterogeneous effects on high-quality development in different types of cities and its transmission mechanism. The research findings show that the low-carbon city pilot policy can significantly promote high-quality economic development in cities and has heterogeneous effects in terms of regional differences, city types, and city scale. The effects are relatively greater in the eastern region, non-resource-based cities, and mega-cities. The low-carbon city pilot policy promotes high-quality economic development through mechanisms such as technological progress effects, resource agglomeration effects, and government action improvement effects. Combining theoretical analysis with empirical results, this study proposes policy recommendations to enhance the effectiveness of the low-carbon city pilot policy in promoting high-quality development.

## 1. Introduction

Promoting the greening and low-carbonization of economic and social development is a key link in achieving high-quality development. The shift of the Chinese economy from high-speed growth to high-quality development is the result of China’s economic development practice and the evolution of development theory. It aims to achieve efficient, fair, and green sustainable development that meets the increasingly growing needs of the people for a better life [1, 2]. To prevent environmental pollution and resource consumption from hindering the improvement of urban development quality, low-carbon governance is a necessary path to achieve high-quality urban development. As one of the largest carbon-emitting countries and a signatory to the Kyoto Protocol, China has formulated long-term plans for low-carbon development and successively identified 6 provinces and 81 cities (regions) as pilot low-carbon cities in 2010, 2012, and 2017. The low-carbon city pilot aims to accelerate the construction of ecological civilization, promote green development, and actively respond to climate change, exploring feasible paths for developing a low-carbon economy and establishing a resource-conserving and environmentally friendly society in urban development, thereby achieving high-quality urban development.

The low-carbon city pilot projects have a certain degree of exploratory nature and do not have a unified model or strong environmental regulatory constraints. The purpose is to form replicable and scalable low-carbon development experiences based on regional characteristics. However, the vigorous development of a low-carbon economy is aimed at achieving sustainable development, with development still being the focal point. The low-carbon city pilot policies achieve sustainable development by promoting energy conservation and emissions reduction, optimizing urban planning, driving the development of green industries, and enhancing environmental awareness and public participation. This creates a sustainable development environment for cities and promotes high-quality urban development. Analyzing the impact of low-carbon city pilot policies on high-quality urban development can further clarify the goals of the pilot, facilitate adjustments to the low-carbon city pilot policies, and carry significant theoretical and practical significance.

## 2. Literature review

The concept and connotation of high-quality development have not been uniformly defined in existing literature, and there are significant differences in how different studies categorize it. The understanding of its connotation is still evolving [3–5]. With a deeper understanding of high-quality development, the measurement methods have been improved. In earlier literature, high-quality development was primarily discussed from the perspective of GDP, using Total Factor Productivity (TFP) as a proxy indicator [6]. While this method reflects factors such as efficiency and structure in economic growth, it fails to reflect the sustainability of economic growth [7]. However, the new development pattern focusing on the domestic circulation as the main body has led to the inclusion of inclusive, green, innovation-driven,and shared concepts in the Green Total Factor Productivity (GTFP), which effectively observes the level of high-quality development in China’s economy [8, 9]. Since the 19th National Congress of the Communist Party of China, "innovation, coordination, greenness, openness, and sharing" have been identified as the connotation of high-quality development, and "the Five Development Concepts" have been used as guidelines for constructing an evaluation system for high-quality development at the urban level, consisting of five dimensions [10], which better meet the requirements and changes of the new era.

The Chinese government has initiated a total of 87 low-carbon city and regional pilot projects in three batches, aiming to drive comprehensive economic transformation through the construction of low-carbon cities. Currently, academic research on the policy evaluation of low-carbon city pilot projects can be broadly classified into two categories.

One category focuses on studying policies designed to reduce urban carbon emissions and energy consumption. The low-carbon city pilot policies are expected to reduce urban carbon emissions and mitigate energy consumption by improving energy efficiency and upgrading industrial structures [11]. These policies are also anticipated to reduce urban smog pollution [12], enhance air quality in pilot areas [13], and have a significant inhibitory effect on electricity consumption [14]. Another category of literature emphasizes the additional benefits beyond energy conservation and emission reduction brought about by low-carbon city pilot policies. These policies encourage pollution-intensive enterprises to reduce emissions through technological innovation, effectively reducing the information asymmetry of green innovation among innovative entities, promoting the progress and spillover of green technologies in cities, and driving industrial structural upgrades [15]. The upgrading of industrial structure can achieve balanced distribution of production factors such as labor and capital across various sectors, thereby improving urban resource allocation [16–18]. Furthermore, urban low-carbon governance contributes to attracting foreign direct investment, bringing advanced management concepts and information technology levels to China’s development, and enhancing the overall technological innovation vitality of cities [19, 20].

However, from the perspective of high-quality urban development, the evaluation of the implementation effects of low-carbon city pilot policies has not received sufficient attention from the academic community. The low-carbon city pilot policies represent a weak incentive and weak constraint environmental regulation [21]. In existing literature, there is no consensus on the impact of environmental regulations on high-quality economic development. According to the "Porter Hypothesis" [22], moderate environmental regulation can promote green technological innovation and facilitate high-quality urban development through the "innovation compensation effect" and "learning effect" [23, 24]. Conversely, the "Compliance Cost Theory" suggests that environmental regulations may increase enterprises’ budgets for pollution control, leading to rising production factor prices, increased enterprise costs, decreased production efficiency, and hindered industrial upgrading, ultimately restraining high-quality urban development [25]. Therefore, it is evident that further research is needed to understand the impact of low-carbon city pilot policies on high-quality urban development.

In conclusion, most studies on low-carbon city pilot policies focus on carbon emissions, green technological innovation, and environmental pollution. This paper focuses on exploring the role of low-carbon city pilot policies in urban high-quality development, with the following main contributions: First, it empirically tests the effects of low-carbon city pilot policies on urban high-quality development. While low-carbon city pilot policies primarily focus on emission reduction, high-quality development embodies the essence and value pursuit of socialism and is crucial for achieving China’s modernization. Incorporating urban high-quality development into the evaluation framework of low-carbon pilot policies provides a new perspective for studying the effects of these policies. Second, it investigates the heterogeneous impacts of low-carbon city pilot policies on different types of cities’ high-quality development. The distribution of pilot cities in terms of regions, types, and sizes varies significantly, and the low-carbon city pilot policies are still being refined. Examining the heterogeneous effects helps different types of cities further evaluate the implemented pilot policies and promote high-quality development more effectively. Third, it explores the mechanisms through which low-carbon city pilot policies influence high-quality development. By discussing the effects from technological progress, resource agglomeration, and government behavior improvement, this research can amplify the effects of pilot policies and provide a theoretical basis for pilot cities to grasp crucial aspects of greening and low-carbonization, promoting high-quality development more effectively, and serving as decision-making references for further policy promotion in pilot cities.

## 3. Case study

Since the mid-19th century, when humans began recording global temperatures, the pace of global climate warming has accelerated in the 20th century, and temperatures continue to rise in the 21st century. Climate change has attracted increasing attention from scholars. As one of the world’s major carbon dioxide emitters, China, as the "largest developing country," faces common challenges for human society and must take active measures to respond to global climate warming and build a community of human destiny. In June 1992, China signed the United Nations Framework Convention on Climate Change (UNFCCC), taking the initiative to shoulder the responsibility for protecting global climate. The Kyoto Protocol legally set the reduction targets for different countries, and China signed the Protocol in 1998. In December 2007, the National Program for Climate Change was promulgated, providing a detailed explanation of China’s current situation in climate change and proposing related policies and measures based on China’s national conditions. It was not only China’s first program on climate change but also the first such program in a developing country. In 2010, the General Office of the National Development and Reform Commission (NDRC) issued the Notice on Carrying out Pilot Projects for Low-Carbon Provinces and Cities, which was first piloted in five provinces and eight cities. In order to continue implementing the spirit of the Notice on Issuing the Twelfth Five-Year Plan for Controlling Greenhouse Gas Emissions by the State Council, in 2012, the second batch of low-carbon city pilot areas was selected, with cities as the main focus and provinces/districts as the secondary focus, piloting low-carbon measures in 29 provinces and cities. In 2017, according to the requirements of the Outline of the Thirteenth Five-Year Plan, the National Plan for Climate Change (2014–2020), and the Work Plan for Controlling Greenhouse Gas Emissions in the Thirteenth Five-Year Plan, the NDRC issued a notice on launching the third batch of national low-carbon city pilot projects, progressively promoting and improving low-carbon efforts, selecting 45 cities (districts/counties) representative and exemplary of low-carbon measures. S1 Table shows the list of low-carbon city pilot policy trial areas.

The "top-down" mode of the low-carbon city pilot policies requires local governments to formulate low-carbon development plans based on local development conditions and resource endowments, and to develop corresponding work plans and supporting policies for low-carbon green development. In terms of technological innovation, local governments actively explore mechanisms for promoting low-carbon technologies, increase investment in the research and development of low-carbon technologies, strengthen cooperation with domestic research institutions, improve application methods for low-carbon technology research and development, and formulate preferential incentives policies for low-carbon technology. Through technological innovation, greenhouse gas emissions can be controlled, production costs can be reduced, and the core competitiveness of low-carbon development can be enhanced, thus strengthening the role of science and technology in supporting the construction of low-carbon cities. In terms of international cooperation, the "internal connection and external aid" strategy is implemented to further expand openness, strengthen cooperation mechanisms with developed countries, learn advanced management concepts from low-carbon diplomacy, actively explore innovative experiences and practices, improve the management capacity for low-carbon development, continuously explore new models for low-carbon city development, and promote greener development in all cities. In terms of financial investment, the investment mechanism of finance at all levels is improved, and the guiding role of financial funds is played well to attract social capital investment in the construction of low-carbon cities. Special funds for low-carbon development are set up, priority is given to guaranteeing funds for low-carbon city construction projects, and tax incentives policies that favor low-carbon development are adopted. Supporting research funding is provided, investment in the research, application, and promotion of low-carbon technologies is increased, and the fund protection system is improved. Enterprise financing channels are expanded to provide strong financial support for enterprise low-carbon technology innovation.

## 4. Theoretical framework and research hypotheses

### 4.1 The logic of the role of low-carbon city pilot policies on the high-quality development of urban economy

The concept of a low-carbon economy emerged against the backdrop of global warming, emphasizing the synergy between low-carbon governance and economic growth. As a loosely constrained environmental regulation with weak incentives, the pilot policies for low-carbon cities require local governments to formulate corresponding low-carbon pilot plans based on their own characteristics. The main objective is to control greenhouse gas emissions by continuously exploring mechanisms and models for low-carbon urban development, promoting the development of low-carbon industries, and facilitating the consumption of green energy. This, in turn, promotes high-quality economic development in cities.Specifically, the pilot policies encourage enterprises to engage in low-carbon technological innovation, generating a "innovation compensation effect" that enhances production efficiency while reducing carbon emissions. This leads to the formation of a low-carbon modern industrial system. Pilot cities actively explore innovative development models and develop a new batch of innovative achievements, such as smart grids, green and low-carbon buildings, and new energy vehicles, which contribute to the green and innovative development of urban economies.The imitation and competition among pilot cities, as well as the exchange and cooperation with developed countries, can enhance knowledge transfer, technological levels, and management capabilities, injecting "fresh blood" into the low-carbon industry and promoting green and open economic development. Considering the uneven regional economic development, the pilot policies for low-carbon cities encourage local governments to formulate green and low-carbon development plans based on local conditions. If proven effective, other cities can explore locally suitable low-carbon development strategies based on the successful experiences of these pilot cities [21]. The cross-regional flow of innovative elements improves the efficiency of the pilots while addressing the needs of slower developing cities, thus promoting coordinated and green economic development.With the popularization of low-carbon lifestyle, initiatives such as green schools, eco-friendly transportation, green buildings, and sustainable consumption improve residents’ quality of life, achieving shared green development in urban economies.

**H1:** Pilot policies for low-carbon cities promote high-quality economic development in urban areas.

### 4.2 Heterogeneity of the effects of low-carbon city pilot policies

Firstly, the pilot policies for low-carbon cities may exhibit regional heterogeneity as cities located in different regions face distinct economic development environments. Coastal cities in the eastern region are more conducive to communication and openness with other countries, have a high level of resource aggregation capacity, and are thus advantageous for policy implementation. The western region, on the other hand, has weaker infrastructure. In order to achieve coordinated regional development, the central government pays greater attention to the development of the western region and allocates more resources to underdeveloped areas, leading to relatively stronger government behavioral improvement effects. Furthermore, the pilot policies for low-carbon cities may encounter heterogeneity in terms of city types. Resource-based cities and non-resource-based cities face different carbon reduction pressures during the construction process of low-carbon pilot cities due to differences in resource types, resource endowments, and utilization levels. Resource-based cities are characterized by the extraction and processing of local natural resources such as minerals and forests, making industrial transformation more challenging and resulting in relatively weaker effects of government administrative intervention. Lastly, the pilot policies for low-carbon cities may exhibit heterogeneity in terms of city scale. The population size of a city reflects its resource aggregation capacity, and people act as the hub and transformer for the free flow of resources. Consumer behavior stimulates the flow of resources [26] and increases per capita disposable income [27]. The larger the city scale, the stronger the resource aggregation effect of the low-carbon pilot policies. Higher population density leads to a richer pool of talent, a stronger foundation for technological innovation, and more prominent effects of technological progress.

**H2:** The pilot policies for low-carbon cities exhibit regional heterogeneity, city type heterogeneity, and city scale heterogeneity.

### 4.3 The mechanism of low-carbon city pilot policies for high-quality urban economic development

#### 4.3.1 Technological progress effect

Cities’ industries heavily rely on non-renewable resources. In order to enhance the innovation capacity of low-carbon technologies in the industrial sector, local governments have formulated pilot policies for low-carbon cities, with a focus on promoting technological innovation. These policies include energy-saving and emission-reducing technologies, clean energy development and utilization technologies, carbon sequestration technologies, carbon capture technologies, and more. By improving the efficiency of resource and energy utilization in the production process, these policies have achieved the decarbonization of the industrial sector and boosted productivity, while continuously advancing supply-side structural reforms and promoting high-quality economic development in cities. Technological progress can extend the industrial chain, increase the added value and depth of product processing, and improve enterprise economic performance while stimulating employment opportunities.Furthermore, the low-carbon policies draw on advanced low-carbon solutions and experiences from both domestic and international sources, and establish platforms for industry-academia-research collaboration in low-carbon technology. Talent attraction policies are implemented in the field of low-carbon technology, with increased efforts to attract talents from both domestic and overseas sources, activating innovative potentials, and leveraging the demographic dividend to enhance confidence in and support the high-quality economic development. Moreover, by relying on research institutions, key technological bottlenecks are overcome, enhancing market competitiveness for enterprises, fostering new models and formats, accelerating industrial transformation, increasing the proportion of knowledge-intensive industries, and promoting high-quality economic development in cities. Therefore, by driving ecological improvement through technological progress, constraints imposed by the low-carbon transition on urban development can be alleviated, achieving harmonious development between ecology and economy, and playing a significant role in promoting high-quality economic development.

#### 4.3.2 Resource agglomeration effect

Scholars who have studied the "pollution haven" hypothesis suggest that extremely strict environmental regulations will impede foreign investment [28, 29]. However, subsidy policies for enterprises under low-carbon city pilot programs can help to reduce investment costs, change the benefits and costs of enterprises in terms of the environment, and promote the investment behavior of foreign companies [19]. In addition, environmental access standards are set for enterprises under these policies, effectively avoiding the "race to the bottom" effect of investment attraction. The proportion of low-carbon industries continues to increase, the industrial structure tends to be rationalized, and economic development becomes greener, all of which are favorable for high-quality economic development in cities. During the low-carbon city pilot process, extensive international cooperation was carried out among various cities, which expanded the cities’ international influence and publicity, improved their resource aggregation capabilities, and resulted in externalities of resource aggregation between neighboring cities. This led to reduced transaction costs, more information spillovers, and economies of scale. Opening up to the outside world is an important prerequisite for enhancing resource aggregation capacity. Modern economies are market-oriented, and their developmental process involves the free flow of resources. The more frequent the exchanges and cooperation between cities and developed countries or regions in low-carbon development, the stronger the cities’ attractiveness and resource aggregation capacity will become. This increases their chances of attracting external funds and advanced technologies, resulting in improved production efficiency and the "pollution halo effect". Therefore, the knowledge sharing and technology spillover effects brought about by resource aggregation can mitigate urban environmental pollution and achieve effective growth in the quality of economic output.

#### 4.3.3 Government behavior improvement effect

The low-carbon city pilot policies emphasize the principles of government guidance, enterprise leadership, and public participation, aiming to leverage the role of government guidance and fully utilize market mechanisms to develop energy-saving and environmental protection industries and promote sustainable urban economic development. Firstly, in the construction of low-carbon cities, the government optimizes the structure of fiscal expenditures, increasing investment in low-carbon technology research and development, and industrial innovation to encourage enterprises to adopt environmentally friendly business practices, enhance ecological awareness, improve circular industry chains, and ultimately achieve the improvement and sustainability of corporate economic benefits, as well as the resilience of the industrial chain, leading to high-quality urban economic development. Secondly, the government will further improve fiscal and tax incentive policies, establish financial subsidies and loan interest discount programs to promote low-carbon development, and transform the economic development model. Finally, local governments will increase research and development subsidies, implement policy-oriented and targeted science and technology policies [30], to some extent alleviating corporate financing constraints, reducing innovation costs for enterprises, and mitigating the social governance costs of pollution, thus achieving a win-win situation for corporate benefits, environmental benefits, and social benefits.

**H3:** the low-carbon city pilot policies promote high-quality urban economic development through the effects of technological progress, resource agglomeration, and improvements in government behavior.

## 5. Study design and data description

### 5.1 Research design

The difference-in-differences (DID) model is one of the key models for policy effect evaluation. Prior to the implementation of low-carbon city pilot policies in China, each city in the country exhibited similar trends in high-quality economic development. This forms the premise of the DID model. From 2010 to 2017, three batches of low-carbon province and city pilot programs have been launched nationwide, covering 6 provinces, 76 prefecture-level cities, 3 counties, as well as the Greater Hinggan Mountains region and Simao District of Pu’er City. This study treats pilot cities as the treatment group and non-pilot cities as the control group. By constructing a DID model, it can be determined whether the changes in the outcome variable of the treatment group are caused by policy impacts. If not, it indicates that the changes in the outcome variable of the treatment group are not due to policy impacts. When the same policy is implemented in the treatment group at the same time, The difference-in-differences model is applicable. When the same policy is implemented in the treatment group at different times, a multi-period difference-in-differences model should be used. Based on data availability, this study examines the policy effects of low-carbon city pilot programs in all cities of the six pilot provinces in the sample and 59 pilot cities.

The multi-period difference-in-differences model is constructed as follows:

$${JJGZL}_{it}=\alpha_{0}+\beta_{1}{LCC}_{it}+\beta_{2}Z_{it}+u_{i}+\gamma_{t}+\varepsilon_{it}$$ (1)

In Model (1), the dependent variable represents the level of high-quality urban economic development in city i at year t. The core explanatory variable is ***LCC***_***it***_,the dummy variable indicating whether city i is a low-carbon pilot city in year t. If city i implemented the low-carbon city pilot policies in year t, the variable is set to 1; otherwise, it is set to 0. The coefficient of interest in this model is the estimated coefficient ***LCC***_***it***_, which represents the effect of the low-carbon city pilot policies on high-quality urban economic development. This coefficient is the central focus of attention as it provides the economic interpretation of the impact of the low-carbon city pilot policies on the level of economic development in cities. In the model, ***Z***_***it***_ represents the control variables that influence high-quality urban economic development. ***u***_***i***_ denotes the city fixed effects, which capture unobserved time-invariant characteristics of individual cities. ***γ***_***t***_ represents the year fixed effects, capturing common time-specific factors affecting all cities. Lastly, ***ε***_***it***_ represents the random error term.

### 5.2 Data description

#### 5.2.1 Variable selection and descriptive statistics

The dependent variable is the high-quality urban economic development (JJGZL). Based on the "Five Development Concepts," a comprehensive evaluation system for high-quality urban economic development in China has been constructed. Following the approach of Wei,M and Li,S [31], the entropy weighting method is employed to calculate the high-quality urban economic development index for Chinese cities. Seventeen indicators were selected from the dimensions of innovation, coordination, greenness, openness, and sharing in a comprehensive and scientific manner. The specific dimensions, indicators, measurement methods, and attributes are shown in Table 1.The core explanatory variable is the dummy variable for low-carbon city pilot policies (LCC). The coefficient of this dummy variable reflects the effect of low-carbon city pilot policies on the level of high-quality urban economic development. The control variables include the level of financial development (Finance), measured by the ratio of outstanding loans of financial institutions to GDP; industrial structure (Industrial), measured by the proportion of value added from the secondary industry to GDP; industrial scale (Scale), measured by the total output value of above-scale enterprises; investment level (Invest), measured by the total fixed asset investment of the whole society; and import-export scale (Open), measured by the total import-export volume. To mitigate the impact of outliers on empirical results, a one-sided winsorization approach was applied to the import-export scale at the 1% level. Descriptive statistics are presented in Table 2.

**Table 1:** Evaluation system of high-quality economic development of China’s cities.

Dimensionality	Specific indicators	Measurement method	Attribute
Innovative development	State of innovation and entrepreneurship	Regional Innovation and entrepreneurship Index	+
	Number of university students per 10,000 people	Number of students in colleges and universities/total population at the end of the year	+
	Output effect of science and education funds	GDP/Science and education expenditure	+
Coordinated development	The level of urban and rural income coordination	Per capita disposable income of urban residents/per capita disposable income of rural residents	-
	Urbanization level	Urban population/Total population at year end	+
	Urban unemployment rate	Number of unemployed urban residents/total population at year-end	-
Green development	Forest coverage rate	Green space/administrative area	+
	Green coverage rate of built-up area	Green coverage rate of built-up area	+
	Urban wastewater discharge	Industrial wastewater discharge /GDP	-
	Urban waste gas emissions	Industrial sulfur dioxide emissions /GDP	-
	Municipal solid waste discharge	Comprehensive utilization rate of general industrial solid waste	+
	Waste disposal rate	Harmless treatment rate of household garbage	+
Open development	Foreign capital dependence	Foreign direct investment /GDP	+
Shared development	Consumer expenditure	Total retail sales of consumer goods/total population at year end	+
	Degree of medical facilities	Number of beds in medical institutions/total population at the end of the year	+
	Completeness of medical services	Number of health workers/total population at year end	+
	Internet penetration	Internet broadband access users 3/ Total population at the end of the year	+

Note:The Statistical Yearbook of China Cities only counted the number of international Internet broadband access users before 2012, so it was the number of international Internet broadband access users from 2005 to 2011.

**Table 2:** Descriptive statistics.

Variable definition	Variable	Obs	Mean	Std.Dev.	Min	Max
High-quality urban Economic development	JJGZL	3,654	12.160	7.823	2.083	64.890
Low carbon pilot policy	LCC	3,654	0.186	0.389	0	1
Financial development level	Finance	3,654	0.322	0.586	0.022	15.170
Industrial structure	Industrial	3,654	0.482	0.109	0.090	0.910
Industrial scale	Scale	3,654	2395	3281	3.143	30710
Investment level	Ivest	3,654	1068	1189	29.490	9404
Import and export scale	Open	3,654	79.530	256.200	0.056	4701

Innovation is the primary driving force for development. This article identifies specific indicators in the dimension of innovation, including the situation of innovation and entrepreneurship, the number of college students per ten thousand people, and the output efficiency of scientific and educational funding. Innovation and entrepreneurship are the "two carriages" that drive high-quality and sustainable economic development [32]. Innovative and entrepreneurial activities contribute to the development of the economy. College students, as the reserve force of urban talent resources, are a vital source of innovation and represent the potential for urban innovation. Technological advancement can increase the proportion of the service industry, while education can optimize human capital. Scientific and educational funding serves as the capital for urban innovation, promoting innovative economic development.

Coordination is an inherent requirement for sustainable and healthy development. Specific indicators in the dimension of coordination include the level of income coordination between urban and rural areas, the level of urbanization, and the urban unemployment rate. With rapid economic growth, the urban-rural divide has become increasingly severe. Coordination between urban and rural areas requires further narrowing of the income gap. As engines of modern economic growth, cities attract rural surplus labor, providing human resources for industrial and service sectors, and driving coordinated urban economic development. Employment absorption serves as a hub for coordinated urban development and the well-being of the people. Unemployment rates of urban residents reflect the coordination of urban economic operations.

Greenness is a necessary condition for sustainable development and an important reflection of people’s pursuit of a better life. Specific indicators in the dimension of greenness include forest coverage rate, built-up area green coverage rate, urban wastewater and gas emissions, urban solid waste emissions, and the rate of harmless treatment of domestic waste. A sound ecological environment benefits all aspects of people’s well-being. Forest coverage rate and built-up area green coverage rate directly reflect the actual level of forest resources and green area occupation in China, as well as the progress of ecological restoration work. The reduction of urban wastewater and gas emissions is an important task in improving the ecological environment and promoting green development. The amount of industrial solid waste can be reduced through recycling, processing, and exchange to extract or convert resources, energy, and other raw materials for utilization, which saves resources and reduces pollution. Various types of waste are important sources of environmental pollution and ecological degradation. Along with economic development, the generation of various types of waste has also increased rapidly. Improving waste treatment efficiency can reduce environmental pollution and promote coordinated development of the economy and ecology.

Openness is the only way to achieve prosperity and development for a country. The specific indicator in the dimension of openness is the degree of reliance on foreign investment. The introduction of foreign capital helps alleviate the problem of capital shortage, brings advanced management concepts, and promotes technological transformation. It enhances economic quality through the exchange of market access for funding and technology, accelerating the upgrading of China’s economy.

Sharing is an essential requirement of socialism with Chinese characteristics. Specific indicators in the dimension of sharing include consumer expenditure, the completeness of public libraries, the completeness of medical facilities, the completeness of medical services, and internet penetration rate. The willingness and level of consumer spending by residents determine whether the achievements of economic development are shared by the people. The completeness of cultural and medical infrastructure is crucial in measuring the health and material well-being of a country’s residents. Improving the completeness of infrastructure helps address people’s livelihood issues and improve their welfare. Meeting the growing demand for a better life among the people requires bridging the gap in infrastructure supply and establishing a foundation for shared economic development. The improvement of internet penetration rate contributes to the development of the digital economy, enabling smart living, working, and learning for residents. It facilitates the construction of next-generation infrastructure, unlocks emerging industries, and provides new momentum for advancing economic and social progress.

According to the descriptive statistics in Table 2, there are 3654 observations in the sample. From 2005 to 2018, the minimum value for high-quality urban economic development is 2.083, while the maximum value is 64.890, with a mean of 12.160. This indicates significant disparities in high-quality urban economic development, with some cities surpassing the average level significantly. Similar situations can be observed in other variables, highlighting the issue of imbalanced and insufficient regional development in China.

#### 5.2.2 Data source

Due to data availability constraints, this study utilizes panel data from 261 prefecture-level cities spanning from 2005 to 2018 as the sample. The dependent variable, the Innovation and Entrepreneurship Index, is sourced from the Enterprise Big Data Research Center at Peking University. The remaining 16 indicators and control variable data are derived from the "China City Statistical Yearbook," "China Regional Economic Statistical Yearbook," EPS data platform, and RESSET macroeconomic database, among others. For a small number of missing values in certain variables within the sample, interpolation methods are employed to complete the data.

## 6. Results and analysis

### 6.1 Benchmark model analysis

Based on the multi-period difference-in-differences model, the impact of low-carbon city pilot policies on high-quality urban economic development is tested, and the results of gradually adding control variables are shown in Table 3.

**Table 3:** Baseline regression results.

Variable	(1)	(2)	(3)	(4)	(5)	(6)
	JJGZL	JJGZL	JJGZL	JJGZL	JJGZL	JJGZL
LCC	0.923***	0.925***	0.935***	0.925***	0.891***	0.811***
	(9.03)	(9.07)	(9.25)	(9.33)	(9.14)	(8.50)
Finance		0.496***	0.230*	0.368***	0.444***	0.510***
		(4.32)	(1.94)	(3.14)	(3.85)	(4.53)
Industrial			-4.847***	-3.940***	-3.903***	-3.377***
			(-7.63)	(-6.28)	(-6.33)	(-5.59)
Scale				0.198***	0.105***	0.080***
				(11.95)	(5.72)	(4.42)
Invest					0.049***	0.050***
					(10.92)	(11.32)
Open						0.027***
						(12.65)
Constant	9.393***	9.252***	11.542***	10.942***	10.818***	10.491***
	(98.31)	(91.88)	(36.48)	(34.86)	(35.04)	(34.64)
Observations	3,654	3,654	3,654	3,654	3,654	3,654
R^2^	0.606	0.608	0.615	0.630	0.643	0.659
Year FE	Yes	Yes	Yes	Yes	Yes	Yes
Urban FE	Yes	Yes	Yes	Yes	Yes	Yes

Note: The t statistic is in parentheses, where

***, **, and * indicate significant levels of 1%, 5%, and 10%, respectively.In order to avoid the small regression coefficient, the industrial scale is divided by 1000, the investment level by 100 and the import and export scale by 10.

Column (1) in Table 3 presents regression results without incorporating any control variables, suggesting that low-carbon city pilot policies foster high-quality urban economic development. As the factors of financial development level, industry structure, industrial scale, investment level, and import-export scale are progressively incorporated from columns (2) to (6), the regression coefficient of the core explanatory variable remains positive at a significant level of 1%. However, there is a noticeable decrease in the coefficient value, from 0.925 to 0.811. This demonstrates a significant enhancement in the level of high-quality economic development in the pilot cities following the implementation of low-carbon city pilot policies, thus validating our first theoretical hypothesis.The boost may be attributed to the active innovation of low-carbon institutional mechanisms in these cities after enacting the policies. Such measures include the development of low-carbon technology and product platforms, localized strategies, promotion of a low-carbon lifestyle and economy, and the establishment of a low-carbon industrial system. These initiatives have collectively improved innovation, coordination, green development, openness, and shared progress, thereby propelling high-quality economic development in the respective cities.

### 6.2 Robustness test

#### 6.2.1 Parallel trend test

The fundamental premise of the multi-period difference-in-differences (DID) method is the parallel trends assumption between the treatment group and the control group prior to policy intervention. The level of high-quality economic development in both pilot and non-pilot cities should exhibit similar trends before the implementation of low-carbon city pilot policies; otherwise, the multi-period DID method might overestimate or underestimate the policy effects.To test the parallel trends, we use the year the policy was first executed (2010) as the baseline and examine the policy’s effects four years prior and eight years post-implementation. The model is constructed as follows:

$${JJGZL}_{it}=\alpha_{0}+\beta_{1}{DID}_{it}^{-4}+\ldots +\beta_{10}{DID}_{it}^{8}+\beta_{11}Z_{it}+u_{i}+\gamma_{t}+\varepsilon_{it}$$ (2)

**Fig 1:**
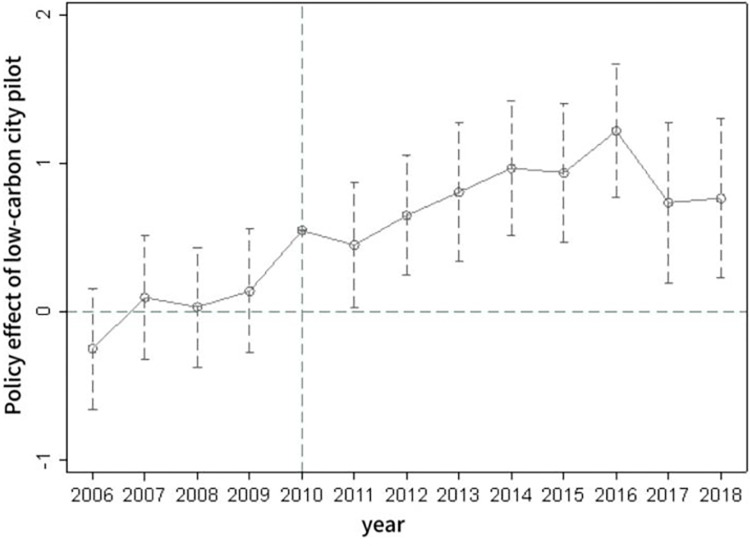
Parallel trends test.

In model (2), DID represents a dummy variable, and its coefficient signifies the policy effect in the corresponding years before and after the pilot phase. Fig 1 illustrates the results of the parallel trends test.

As can be deduced from Fig 1, there is no significant difference from zero in terms of the impact of low-carbon city pilots on high-quality urban economic development prior to the implementation of the low-carbon city pilot policies. This verifies that the treatment and control groups satisfy the parallel trends assumption, and the increase in the level of high-quality urban economic development estimated earlier is indeed attributable to the low-carbon city pilot policies.From a dynamic perspective, policy effects are present from the first to the eighth year following the implementation of the low-carbon city pilot policies, with these effects intensifying annually within the six years post-implementation. This reflects an ongoing accumulation of the impacts of the low-carbon city pilot policy, implying strong sustainability in its role in enhancing high-quality urban economic development. The influence of the low-carbon city pilot policy on the level of high-quality urban economic development appears to strengthen over time.Therefore, it is necessary to continue promoting the construction of low-carbon cities, expand the scope of low-carbon city pilots, and drive the advancement of high-quality urban economic development.

#### 6.2.2 Propensity score matching differential model test

To mitigate self-selection bias, we draw on the methods of Heckman [33, 34] and construct a propensity score matching difference-in-differences (PSM-DID) model by combining propensity score matching (PSM) with the difference-in-differences (DID) method. In this study, we adopted 1:1 nearest neighbor matching, selecting five control variables—financial development level, industry structure, industrial scale, investment level, and import-export scale—as covariates for pairing cities in the treatment and control groups.To visually assess the matching effectiveness and evaluate the appropriateness of this method, we depict the kernel density graphs before and after matching.

**Fig 2:**
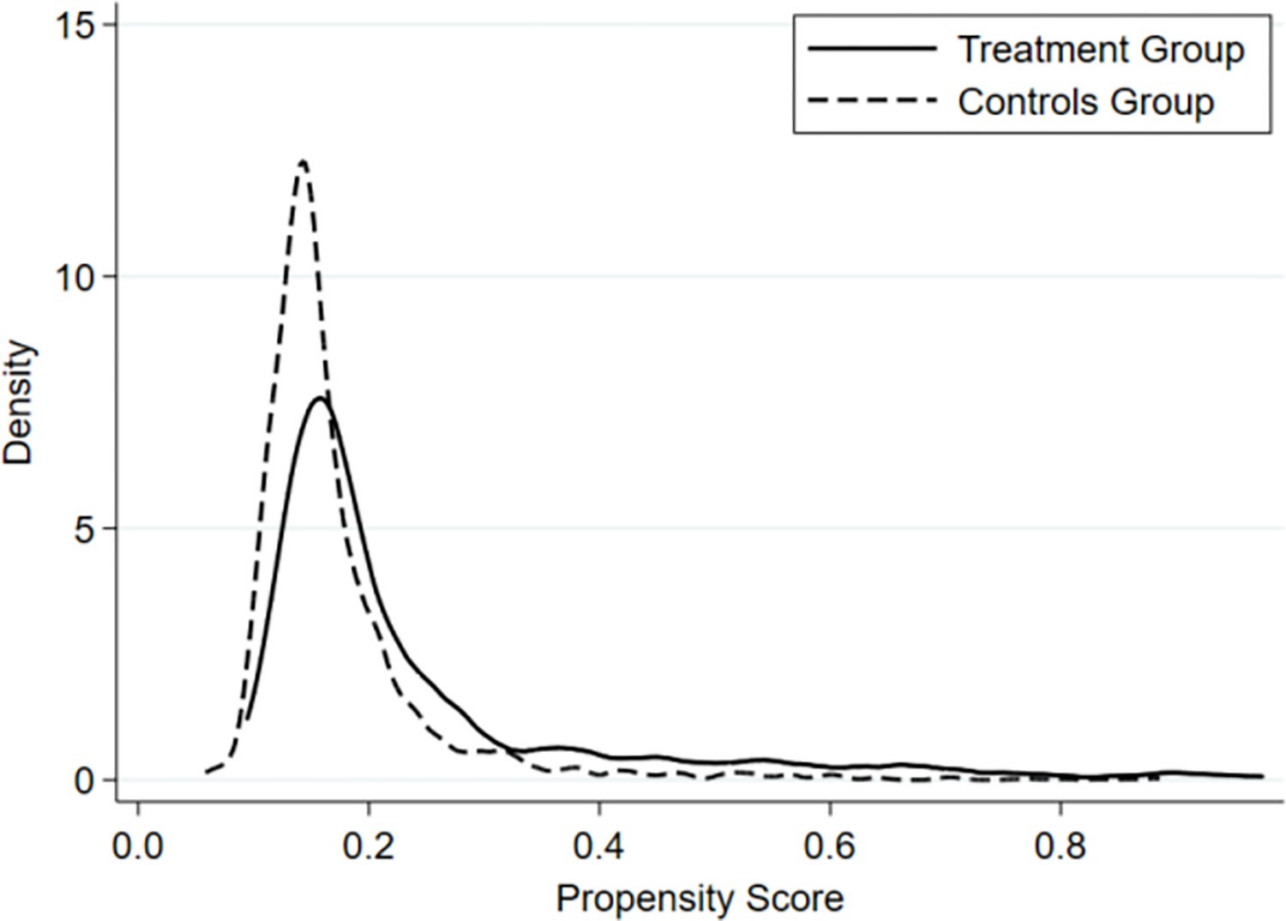
Pre-matching kernel density plot.

**Fig 3:**
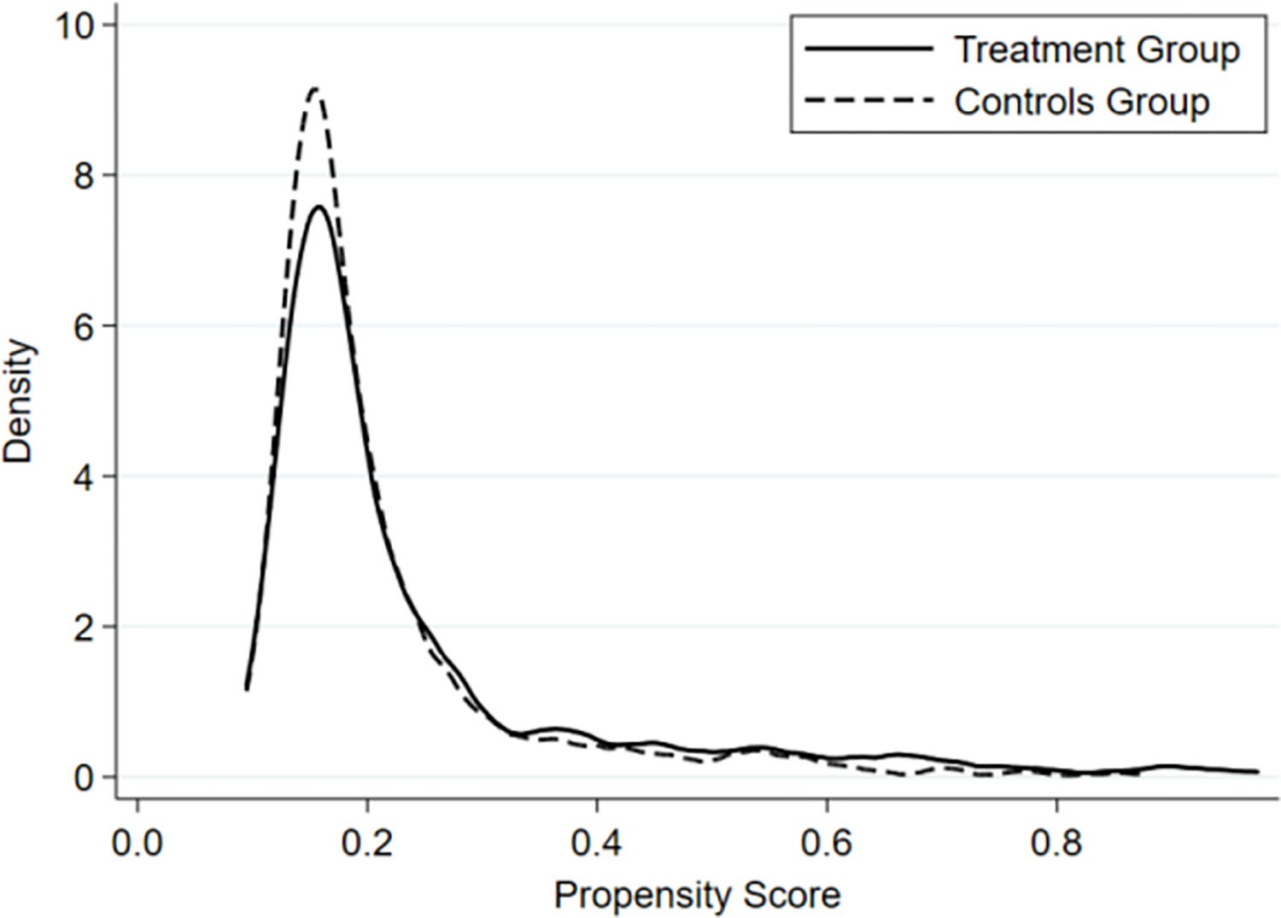
Post-matching kernel density plot.

Figs 2 and 3 are the kernel density graphs before and after matching, respectively, which visually demonstrate the effect of the match. In these figures, the horizontal axis represents the propensity index, and the vertical axis represents density. The dashed line denotes the control group, while the solid line illustrates the treatment group. Fig 2 indicates a substantial disparity in skewness and kurtosis between the treatment group and control group before matching, with a relatively small overlapping area. Conversely, Fig 3 shows a noticeable increase in the overlapping area between the control and treatment groups after matching compared to Fig 2. This demonstrates that near-neighbor matching can mitigate the issue of sample self-selection to a certain extent, thereby reducing bias in the estimation of the DID model.

Utilizing Model (1) and adopting the PSM-DID model, we further examine the impact of low-carbon city pilot policies on high-quality urban economic development. The empirical results are displayed in Table 4.

**Table 4:** PSM-DID test.

Variable	(1)	(2)	(3)	(4)	(5)	(6)
	JJGZL	JJGZL	JJGZL	JJGZL	JJGZL	JJGZL
LCC	0.903***	0.909***	0.904***	0.922***	0.999***	0.961***
	(4.17)	(4.21)	(4.22)	(4.40)	(4.80)	(4.84)
Finance		0.319*	0.077	0.172	0.228	0.322*
		(1.85)	(0.43)	(0.97)	(1.30)	(1.91)
Industrial			-5.254***	-5.011***	-5.132***	-4.176***
			(-4.07)	(-3.97)	(-4.11)	(-3.49)
scale				0.197***	0.134***	0.057*
				(6.25)	(3.92)	(1.68)
Invest					0.033***	0.032***
					(4.43)	(4.45)
Open						0.063***
						(8.98)
Constant	10.340***	10.232***	12.508***	12.097***	12.077***	11.406***
	(34.73)	(33.77)	(19.71)	(19.38)	(19.57)	(19.19)
Observations	1,092	1,092	1,092	1,092	1,092	1,092
R^2^	0.606	0.608	0.616	0.633	0.641	0.673
Year FE	Yes	Yes	Yes	Yes	Yes	Yes
Urban FE	Yes	Yes	Yes	Yes	Yes	Yes

Note: The t statistic is in parentheses, where

***, **, and * indicate significant levels of 1%, 5%, and 10%, respectively.

After the 1:1 nearest neighbor matching, the number of observations is reduced from the original 3654 to 1092. Column (1) represents the regression results without incorporating any control variables, indicating that the low-carbon city pilot policies foster high-quality urban economic development. Columns (2) through (6) sequentially add control variables, and the regression coefficient of the key explanatory variable remains positive at the 1% significance level. The empirical results of the propensity score matching difference-in-differences (PSM-DID) model show little difference from the base regression results, and the qualitative conclusions are consistent. This underscores the robustness of the findings that low-carbon city pilot policies promote high-quality urban economic development.

#### 6.2.3 Exclude other policy influences

During the low-carbon city policy pilot phase, there may be other policies impacting high-quality urban economic development, such as the innovative city pilot policy (innov). To eliminate the potential effects of this policy on our estimations, we introduce a dummy variable representing the implementation of the innovative city pilot policy into Model (1).The regression results are shown in Columns (1) and (2) of Table 5. Column (1) presents the empirical results without incorporating any control variables, while Column (2) introduces control variables. Both the coefficients of the low-carbon city pilot policy and the innovative city pilot policy are significantly positive, consistent with our expectations. Even when controlling for other policy interferences, the low-carbon city pilot policy continues to significantly promote high-quality urban economic development.

**Table 5:** Robustness test.

	Other policies		Substitution variable	
	(1)	(2)	(3)	(4)
Variable	JJGZL	JJGZL	Per_GDP	Per_GDP
LCC	0.820***	0.771***	0.730***	0.608***
	(8.36)	(8.18)	(8.24)	(7.79)
Innov	2.412***	1.420***		
	(17.47)	(9.71)		
Finance		0.546***		-0.224**
		(4.91)		(-2.43)
Industrial		-2.578***		-3.455***
		(-4.29)		(-6.99)
scale		0.067***		0.156***
		(3.73)		(10.54)
Invest		0.038***		0.048***
		(8.49)		(13.29)
Open		0.022***		0.028***
		(10.53)		(16.33)
Constant	9.393***	10.176***	1.568***	2.856***
	(102.63)	(33.86)	(18.94)	(11.52)
Observations	3,654	3,654	3,654	3,654
R^2^	0.638	0.668	0.604	0.695
Year FE	Yes	Yes	Yes	Yes
Urban FE	Yes	Yes	Yes	Yes

Note: The t statistic is in parentheses, where

***, **, and * indicate significant levels of 1%, 5%, and 10%, respectively.

#### 6.2.4 Replace the explained variable

To eliminate estimation bias resulting from measurement errors in the dependent variable, we change the measurement index of the dependent variable based on Model (1). The dependent variable in the original model refers to the high-quality urban economic development index measured by the entropy method. Following the approach of Chen,S,Y and Chen,D,K, et al. [35], we substitute it with GDP per capita as a measure of high-quality urban economic development.The regression results are shown in Columns (3) and (4) of Table 5. Column (3) doesn’t include any control variables, whereas Column (4) does. The estimated coefficients for the low-carbon city pilot policy in both columns are still significantly positive at the 1% level. This demonstrates the robustness of the result that the low-carbon city pilot policy promotes high-quality urban economic development.

## 7. Further discussion

### 7.1 Pilot effect analysis

The low-carbon city pilot policy has significant implications in exploring a low-carbon development path commensurate with high-quality development, spreading from some pilot cities to more cities, distilling successful experiences, and realizing the intent of multiple batches of pilot policies. In this paper, the policy effects of different batches are tested separately, and the empirical results are shown in Table 6.

**Table 6:** Analysis of pilot effects.

Variable	(1)	(2)	(3)
	JJGZL	JJGZL	JJGZL
LCC	0.617***	0.893***	1.107***
	(5.19)	(5.69)	(5.45)
Finance	0.421***	0.277***	0.315***
	(3.57)	(2.68)	(2.98)
Industrial	-2.700***	-2.057***	-2.303***
	(-4.12)	(-3.17)	(-3.55)
Scale	0.078***	-0.030	-0.010
	(3.34)	(-1.61)	(-0.51)
Invest	0.041***	0.042***	0.051***
	(7.05)	(8.19)	(10.14)
Open	0.064***	0.025***	0.024***
	(15.87)	(7.90)	(6.22)
Constant	9.339***	9.212***	9.583***
	(28.62)	(28.34)	(29.60)
Observations	2,884	2,422	2,478
R^2^	0.635	0.672	0.678
Year FE	Yes	Yes	Yes
Urban FE	Yes	Yes	Yes

Note: The t statistic is in parentheses, where

***, **, and * indicate significant levels of 1%, 5%, and 10%, respectively.

Columns (1) to (3) in Table 6 represent the effects of the first, second, and third batches of policy pilots respectively, with all three batches showing significantly positive effects at the 1% level. This implies that each batch of pilot policies effectively elevated the level of high-quality urban economic development.Examining the coefficient sizes, the policy coefficients for the first, second, and third batch pilot cities are 0.617, 0.839, and 1.107 respectively, reflecting a gradually increasing trend. This indicates the scaling effect of the low-carbon city pilot policy, with a successful pilot performance and strong "demonstration effect" in previous batches accumulating valuable replicable experiences for subsequent batches. Consequently, the three batches of low-carbon city pilot policies all demonstrated significant effects.

### 7.2 Heterogeneity effect analysis

#### 7.2.1 Regional heterogeneity effect analysis

According to the grouping by the National Bureau of Statistics, we divided the sample into four subsamples. The eastern region includes Beijing, Tianjin, Hebei, Shanghai, Jiangsu, Zhejiang, Fujian, Shandong, Guangdong, and Hainan. The central region contains Shanxi, Anhui, Jiangxi, Henan, Hubei, and Hunan. The western region comprises Inner Mongolia, Guangxi, Chongqing, Sichuan, Guizhou, Yunnan, Tibet, Shaanxi, Gansu, Qinghai, Ningxia, and Xinjiang. The northeast region includes Liaoning, Jilin, and Heilongjiang.Development disparities among regions may lead to heterogeneity in the effects of the low-carbon city pilot policy. The regression results are displayed in Table 7.

**Table 7:** Regional heterogeneity.

Variable	Eastern Region	Central Region	Western Region	Northeast Region
	(1)	(2)	(3)	(4)
	JJGZL	JJGZL	JJGZL	JJGZL
LCC	0.718***	0.175	0.620***	0.127
	(3.45)	(1.28)	(4.04)	(0.79)
Finance	3.075***	9.412***	0.133	-0.509
	(7.19)	(15.25)	(1.20)	(-1.48)
Industrial	-6.372***	1.160	-4.365***	0.591
	(-3.54)	(1.22)	(-4.98)	(0.58)
Scale	0.053*	-0.025	0.018	-0.008
	(1.69)	(-0.80)	(0.48)	(-0.16)
Invest	0.020*	0.044***	0.064***	0.045***
	(1.94)	(7.26)	(8.19)	(4.69)
Open	0.067***	0.114***	0.013***	0.010***
	(11.62)	(11.36)	(2.75)	(5.83)
Constant	13.861***	5.790***	9.381***	8.746***
	(14.48)	(12.06)	(22.34)	(17.41)
Observations	1,078	1,064	1,078	434
R^2^	0.712	0.766	0.689	0.740
Year FE	Yes	Yes	Yes	Yes
Urban FE	Yes	Yes	Yes	Yes

Note: The t statistic is in parentheses, where

***, **, and * indicate significant levels of 1%, 5%, and 10%, respectively.

From columns (1) to (4) in Table 7, it can be observed that the coefficients of the low-carbon city pilot policy in columns (1) and (3) are significantly positive, while the ones in columns (2) and (4) are not significant. This signifies that the low-carbon city pilot policy has markedly fostered the level of high-quality economic development in cities in the eastern and western regions, but it has not had an impact on those in the central and northeastern regions.A potential explanation for these outcomes is that in the eastern region, with the progress of high-tech industries and the advancement of industrial transformation and upgrading, cities are more capable of fostering low-carbon technological innovation and low-carbon industrial systems. They have significant superiority in facets of eco-environmental governance technology and experience, and can balance the needs of carbon emission reduction and economic development. In contrast, the western region, which has lagged in development and faces significant development pressure, places a higher value on the new development opportunities brought by the low-carbon city pilot policy and is better equipped to utilize policy support for advancement. In recent years, as the state has gradually shifted focus to the development of the western region by implementing a series of measures such as the western development, a foundation has been laid for reducing regional disparities. As the western region has a weak foundation, low energy consumption, and ample room for the application of low-pollution eco-technologies, the implementation of the low-carbon city pilot policy can yield rapid and favorable responses.The central region, which is in the middle range of the national emission reduction and development, is more likely to simply copy and paste policies, and fails to issue corresponding policies that align well with its local characteristics, resulting in less effective policy outcomes. The proportions of secondary industries in the central and northeastern regions are positively affected, while there is a negative effect in the eastern and western regions. This indicates a long-term “carbon-lock-in” effect in the cities in the central and northeastern regions, which has led to a lesser impact of low-carbon policies on high-quality economic development in these cities.

#### 7.2.2 Urban type heterogeneity effect analysis

According to the "National Sustainable Development Plan for Resource-based Cities (2013–2020)," the sample cities were divided into resource-based cities and non-resource-based cities, and empirical tests were conducted on the two sub-samples. The regression results for columns (1) to (2) in Table 8 show that the core explanatory variable in column (1) is not significant. This indicates that the low-carbon city pilot policy has not had a significant impact on the high-quality economic development of resource-based cities. In contrast, the coefficient in column (2) is significant and positive, which suggests that the low-carbon city pilot policy has significantly promoted the high-quality economic development of non-resource-based cities.This can be explained as follows: Cities with abundant resource endowments rely heavily on high-energy consumption and high-emission resource-based industries. In the early stages, these cities had low utilization rates of resources but high pollution levels. After the implementation of the low-carbon city pilot policy, the pilot resource-based cities faced the difficult situation of having a large carbon emission base, making the task of reducing emissions even more challenging [36]. Enterprises are required to adopt cleaner and more environmentally friendly technologies and low-carbon technologies, and environmental regulation has increased their production costs. It is difficult for enterprises to transform solely on their own. Local governments in resource-based cities often have limited financial resources and cannot provide sufficient funding to support enterprise transformation and development. It is challenging to achieve coordinated ecological and economic development in the short term. In contrast, the development of non-resource-based cities relies on well-developed industrial structures and a good foundation for innovation, which is conducive to enhancing the level of high-quality economic development.

**Table 8:** Urban heterogeneity.

Variable	Resource-oriented	Non-resource-based	Small and medium-sized city	Big city	Megacity
	(1)	(2)	(3)	(4)	(5)
	JJGZL	JJGZL	JJGZL	JJGZL	JJGZL
LCC	-0.021	0.894***	-0.237	0.544***	1.020***
	(-0.19)	(6.57)	(-0.21)	(5.37)	(7.66)
Finance	1.581***	0.155	-0.086	0.839***	1.325
	(9.20)	(1.08)	(-0.25)	(2.65)	(1.31)
Industrial	2.293***	-8.143***	1.758	-2.283***	-5.363***
	(3.71)	(-8.67)	(0.32)	(-3.74)	(-4.98)
Scale	0.040	0.068***	-0.943	0.145***	0.038*
	(1.57)	(2.86)	(-1.01)	(6.02)	(1.86)
Invest	0.020***	0.054***	0.253***	0.038***	0.061***
	(3.67)	(8.98)	(3.57)	(5.80)	(12.53)
Open	0.006	0.024***	-0.004	0.047***	0.016***
	(0.70)	(9.45)	(-0.12)	(11.78)	(7.85)
Constant	5.884***	13.813***	9.638***	9.447***	12.158***
	(17.63)	(30.57)	(2.84)	(28.22)	(23.01)
Observations	1,470	2,184	153	2,240	1,263
R^2^	0.732	0.664	0.535	0.696	0.777
Year FE	yes	yes	yes	yes	yes
Urban FE	yes	yes	yes	yes	yes

Note: The t statistic is in parentheses, where

***, **, and * indicate significant levels of 1%, 5%, and 10%, respectively.

#### 7.2.3 Urban size heterogeneity effect analysis

According to the 2014 notice from the State Council of China on adjusting the criteria for urban scale classification, the sample was divided into three sub-samples: small and medium-sized cities (with a population of less than 1 million residents), large cities (with a population between 1 million and 5 million residents), and mega-cities (with a population of over 5 million residents). The regression results for columns (3) to (5) in Table 8 are presented. In column (3), the coefficient of the core explanatory variable is not significant. However, in columns (4) and (5), which correspond to large cities and mega-cities respectively, the regression coefficients are significant and positive at the 1% level. This confirms the existence of heterogeneity in the impact of the low-carbon city pilot policy on the high-quality economic development of cities based on their scale, with larger cities experiencing a more pronounced effect from the policy.The underlying reasons for this can be explained as follows: Larger cities tend to have higher per capita income levels, which leads to greater enthusiasm among residents for adopting low-carbon lifestyles. Consequently, the low-carbon pilot policy can more rapidly integrate into these cities. Furthermore, larger cities possess a stronger population base, and their governments typically place greater emphasis on cultivating residents’ low-carbon awareness. As a result, residents’ consumption concepts and preferences are optimized, making them more inclined to choose low-carbon products and support low-carbon enterprises in their daily lives.

In summary, the low-carbon city pilot policy has exhibited regional heterogeneity, urban typology heterogeneity, and urban scale heterogeneity in terms of its impact on high-quality economic development. These findings have confirmed the theoretical hypothesis 2 presented earlier.

### 7.3 Study on the mechanism of action

Based on the objectives and existing literature of the low-carbon city pilot policy, it is likely to enhance the level of high-quality economic development in cities through the effects of technological progress, resource agglomeration, and government behavior improvement.

The "Porter Hypothesis" theory suggests that environmentally sound policies can stimulate an "innovation compensation" effect, which is beneficial for promoting the upgrade of low-carbon environmental technologies and offsetting the "environmental compliance costs" for businesses, thereby facilitating their sustainable development. The low-carbon city pilot policy may align with the Porter Hypothesis, as it can promote high-quality economic development through the effects of technological progress [37]. The game between local governments in environmental policies and international cooperation can influence foreign direct investment inflows. Due to variations in the stringency of environmental regulations across different regions, neighboring areas surrounding regions implementing environmental regulations may choose to weaken such regulations to attract foreign investment [38]. Therefore, the low-carbon city pilot policy may generate resource agglomeration effects, breaking away from the "pollution haven" hypothesis of previous environmental regulations and promoting high-quality economic development in cities. As market-oriented reforms progress, the role of the government in the market and enterprises becomes increasingly defined, and the government’s means in low-carbon city construction are gradually maturing. The low-carbon city pilot policy may stimulate high-quality economic development through the effects of government behavior improvement.

To examine the role of these three mechanisms, the following econometric model can be established:

$$Y_{it}=\alpha_{0}+\beta_{1}{LCC}_{it}+\beta_{2}Z_{it}+u_{i}+\gamma_{t}+\varepsilon_{it}$$ (3)

In Model (3), Y represents the mediating variables: technological progress effect (Grpat), resource agglomeration effect (FDI), and government behavior improvement effect (Gov). The technological progress effect is measured by the number of green patent applications. Green patent applications are classified into two types: green invention patents (Grinvpat) and green utility model patents (Grutypat). Data for green patent applications are sourced from the State Intellectual Property Office of the People’s Republic of China, and the classification is based on the "International Patent Classification Green Inventory" published by the World Intellectual Property Organization (WIPO) in 2010. The resource agglomeration effect is measured by the ratio of actual utilized foreign investment to the total population at the end of the year. The government behavior improvement effect is measured by the proportion of scientific and technological expenditure to the general public budget expenditure of the government. The regression results are presented in Table 9.

**Table 9:** Test of mechanism of action.

Variable	(1)	(2)	(3)	(4)
	Grinvpat	Grutypat	FDI	Gov
LCC	212.033***	140.202***	18.991**	0.154***
	(8.55)	(7.56)	(2.57)	(2.93)
Finance	-136.027***	-116.829***	-9.748	-0.129**
	(-4.64)	(-5.33)	(-1.12)	(-2.09)
Industrial	-785.994***	-816.466***	-81.381*	-0.192
	(-5.01)	(-6.96)	(-1.74)	(-0.58)
Scale	-1.396	-1.971	6.318***	0.058***
	(-0.30)	(-0.56)	(4.52)	(5.88)
Invest	19.191***	14.185***	4.201***	0.015***
	(16.68)	(16.50)	(12.25)	(6.05)
Open	8.864***	7.069***	-0.450***	0.005***
	(16.10)	(17.18)	(-2.74)	(4.33)
Constant	329.221***	364.694***	84.524***	0.300*
	(4.18)	(6.20)	(3.60)	(1.80)
Observations	3,654	3,654	3,654	3,654
R^2^	0.331	0.380	0.194	0.290
Year FE	Yes	Yes	Yes	Yes
Urban FE	Yes	Yes	Yes	Yes

Note: The t statistic is in parentheses, where

***, **, and * indicate significant levels of 1%, 5%, and 10%, respectively.

The columns (1) to (2) in Table 9 present the empirical results of the technological progress effect, while column (3) shows the empirical results of the resource agglomeration effect, and column (4) presents the empirical results of the government behavior improvement effect. The positive significance of the results in columns (1) to (4) indicates that the implementation of the low-carbon city pilot policy has significantly promoted the technological progress effect, resource agglomeration effect, and government behavior improvement effect in the pilot areas. After the policy implementation, these three channels have played a significant role in promoting economic development.Local governments have strengthened environmental regulation, established greenhouse gas emission control responsibility systems, and increased the production threshold and pollution emission cost pressure for high-polluting enterprises in pilot areas. This has prompted these enterprises to actively engage in green technology innovation, effectively improving resource utilization efficiency, and promoting high-quality economic development in cities. To alleviate financing constraints on enterprises and encourage and support them in technology research and development and innovation, the government has increased funding input, innovated tax incentives policies, and increased subsidies, thereby improving the energy-saving and emission-reduction environment for enterprises. The benefits obtained by enterprises from the low-carbon city pilot policy are greater than the costs they incur, which has attracted foreign investment, brought in capital inflows, and expanded economic scale, thus contributing to the improvement of the level of high-quality economic development. In summary, the technological progress effect, resource agglomeration effect, and government behavior improvement effect are important pathways for the low-carbon city pilot policy to promote high-quality economic development in cities, which validates the theoretical hypothesis 3 mentioned earlier.

## 8. Conclusions and policy implications

The implementation of a weak-constraint environmental regulation model in low-carbon city construction pilot projects has captured a key aspect of high-quality economic development. This study selected 261 prefecture-level cities as samples from 2005 to 2018. The entropy weight method was used to calculate the index of high-quality economic development in cities. A multi-period difference-in-differences model was employed to empirically analyze the impact, heterogeneity effects, and mechanisms of low-carbon city pilot policies on high-quality economic development. The research findings are as follows: Firstly, low-carbon city pilot policies can significantly promote high-quality economic development in cities, and they exhibit heterogeneity effects based on regional differences, city types, and city sizes, with the greatest effects observed in eastern regions, non-resource-based cities, and mega-cities. Secondly, low-carbon city pilot policies promote high-quality economic development through mechanisms such as technological progress, resource agglomeration, and improvement in government behavior.

These conclusions provide empirical evidence for further exploration of low-carbon city construction and the promotion of high-quality economic development. Based on this, the following policy recommendations are proposed:

Accelerate the promotion of experiences in low-carbon city construction. Low-carbonization and greenization are crucial for achieving high-quality economic development. It is necessary to summarize the successful experiences and shortcomings of the three batches of low-carbon city construction pilot projects conducted at various levels nationwide, with a particular focus on their role in promoting high-quality economic development. Efforts should be made to select a new batch of low-carbon city construction pilot cities as soon as possible, taking into account the differences among city categories. The number and types of pilot cities should be continuously expanded to accumulate experience for nationwide promotion of low-carbon city construction, thereby accelerating China’s economic transformation, upgrading, and high-quality development.

Develop differentiated incentive and constraint policies for low-carbon city construction. The effects of low-carbon city pilot policies on promoting high-quality economic development exhibit heterogeneity. The analysis of heterogeneity effects should consider objective conditions such as location, development foundation, and city size, as well as subjective attitude differences among different types of cities towards low-carbon city construction. The existing pilot policies belong to a weak-constraint environmental regulation, mainly accelerating construction through incentives, without imposing strict constraints on the behaviors of local pilot cities. Such policy design is conducive to local governments adapting to their own characteristics and conducting more flexible low-carbon city construction pilots, which has played a significant role in facilitating the smooth implementation of pilot projects. However, after more than a decade of exploration, the power dynamics between the central and local governments have changed. Therefore, based on the commonalities and differences among different types of low-carbon construction pilot cities, a system of continuous incentives should be established, incorporating assessment indicators, and implementing classified management systems. This approach will not only leverage the flexibility of local governments but also overcome their inertia, preventing some local governments from being passive.

Strengthen optimization of key aspects of low-carbon city pilot policies. Local governments should strengthen the intermediate mechanisms of technological progress, resource agglomeration, improvement in government behavior, and other key factors, while optimizing and adjusting pilot policies. Low-carbon city pilot policies should not only emphasize the rational utilization of market mechanisms to induce technological innovation but also utilize economic policies to increase support for technological innovation. As the scope of low-carbon city pilots expands, efforts should be made to continuously optimize the business environment to attract foreign investment, avoiding the "race to the bottom" effect caused by strict environmental regulations in the past. Additionally, greater emphasis should be placed on international cooperation to obtain construction funds, improve development conditions, enhance the image of cities, and enhance the ability of local areas to aggregate resources. As an important player in low-carbon city pilot policies, the government should pay more attention to the guiding role of government funds, formulate reasonable tax incentive policies, and provide appropriate subsidies to energy-saving and environmental protection enterprises.

## Supporting information

S1 File Data. (DTA)

S1 Table List of pilot policies for low-carbon cities. (DOCX)

S2 Table Some local policy documents for low-carbon city pilots. (DOCX)

## References

[1] Hong Y. Evolution of Development Concepts and Corresponding Economic Theories since the Reform and Opening-Up Policy: A Discussion on the Theoretical Origins of High-Quality Development. *Economic Perspectives*. 2019;(08), 10–20. Chinese.

[2] Zhang J., Hou Y., Liu P., He J., & Zhuo X. Target Requirements and Strategic Paths for High-Quality Development. *Journal of Management World*. 2019;(07), 1–7. Chinese.

[3] Zhang Z., & Bi Z. Economic High-Quality Development. *Economic Research Journal*. 2022;(04), 21–32. Chinese.

[4] Gao P., Yuan F., Hu H., & Liu X. Driving Forces, Mechanisms, and Governance of High-Quality Development. *Economic Research Journal*. 2020;(04), 4–19. Chinese.

[5] Yang Y., & Zhang P. Logic, Measurement, and Governance of China’s Economic High-Quality Development. *Economic Research Journal*. 2021;(01), 26–42. Chinese.

[6] He X., & Shen K. Modern Economic System, Total Factor Productivity, and High-Quality Development. Shanghai *Economic Research Journal*. 2018;(6), 25–34. Chinese.

[7] Chen H., & Luo L. The Impact and Spatial Effects of Environmental Regulations on Economic High-Quality Development: Based on the Perspective of Industry Structure Transformation. *Journal of Beijing Institute of Technology(Social Sciences Edition)*. 2021;23(06), 27–40. Chinese.

[8] Yu Y., Yang X., & Zhang S. Research on the Spatial-Temporal Conversion Characteristics of China’s Economic Development from High-Speed Growth to High-Quality Development. *Journal of Quantitative & Technological Economics*. 2019;36(06), 3–21. Chinese.

[9] Li H., & Dong Y. Exploring the Level and Differences of China’s High-Quality Economic Development: Based on the Inclusive Green Total Factor Productivity Perspective. *Journal of Finance and Economics*. 2021;47(08), 4–18. Chinese.

[10] Zhou K., & Yu L. The Impact of Intellectual Property Demonstration City Pilot on China’s High-Quality Economic Development: Quasi-Natural Experiment Evidence Based on Double Difference Method. *Journal of Yunnan University of Finance and Economics*. 2021;37(11),13–29. Chinese.

[11] Zhou D., Zhou F., & Wang X. Evaluation and Mechanism Analysis of the Impact of Low-Carbon Pilot Policies on Urban Carbon Emission Performance. *Resources Science*. 2019;41(03), 546–556. Chinese.

[12] Wang H., & Shi D. Can New Urbanization Help Alleviate Smog Pollution? Evidence from Low-Carbon City Construction. *Journal of Shanxi University of Finance and Economics*. 2019;41(10), 15–27. Chinese.

[13] Gehrsitz M. The Effect of Low Emission Zones on Air Pollution and Infant Health.*J*.*Journal of Environmental Economics and Management*. 2017;83(5):121–144. Chinese.

[14] Li S. The Impact of Low-Carbon City Pilot Policies on Electricity Consumption Intensity: An Analysis Based on Synthetic Control Method. *Urban Problems*. 2018; (07), 38–47. Chinese.

[15] Shao S., & Li J. Can Low-Carbon City Pilot Policies Promote Green Technology Progress? An Examination Based on Progressive Double Difference Model. *Journal of Beijing Institute of Technology(Social Sciences Edition**)*. 2022;24(04), 151–162. Chinese.

[16] Xu J., & Cui J. Low-Carbon Cities and Green Technology Innovation of Enterprises. *China Industrial Economics*. 2020;(12), 178–196. Chinese.

[17] Wang F., & Ge X. (2022). Does Low-Carbon Transformation Affect Employment? Empirical Evidence from Low-Carbon City Pilots. *China Industrial Economics*. 2022;(05), 81–99. Chinese.

[18] Zhao Z., Cheng Z., & Lü D. Does National Low-Carbon Strategy Increase Total Factor Productivity of Enterprises? Evidence from Quasi-Natural Experiment of Low-Carbon City Pilots. *Industrial Economics Research*. 2021;(06), 101–115. Chinese.

[19] Gong M., Liu H., & Jiang X. The Impact of China’s Low-Carbon Pilot Policies on Foreign Direct Investment: A Study Based on Evidence from Low-Carbon Cities. *China Population*,*Resources and Environment*. 2019;29(06), 50–57. Chinese.

[20] Zhou Z., Deng L., Xiao H., Wu S., & Liu W. The Impact of Foreign Direct Investment on China’s High-Quality Economic Development: An Analysis Based on Index DEA and Panel Quantile Regression. *Chinese Journal of Management Science*. 2022;30(05), 118–130. Chinese.

[21] Zhuang G. Policy Design Logic of China’s Low-Carbon City Pilot. *China Population*, *Resources and Environment*. 2020;30(03), 19–28. Chinese.

[22] Porter M E. America’s green strategy.*Scientific Amercian*. 1991;264(4):168.

[23] Brunnermeier S.B, Cohen. “Determinants of Environmental Innovation in U S Manufacturing Industries”.*Journal* of Environmental *Economics and Management*. 2003;45(4):278–293.

[24] Wang B., & Liu G. Energy Conservation and Emission Reduction with China’s Green Economic Growth: A Perspective from Total Factor Productivity. *China Industrial Economics*. 2015;(5), 57–69. Chinese.

[25] Jaffe A.B., Palmer K. Environmental Regulation and Innovation:A Panel Data Study. *Review of Economics & Statistics*. 1997;4,610–619.

[26] Guo Q., Xu Y., & Liu C. Factors and Formation Mechanism of Resource Agglomeration Capability in the Central Urban Cluster of the Yangtze River. *China Population*, *Resources and Environment*. 2018;28(02), 151–157. Chinese.

[27] Cao X., & Gao Y. Did the Low-carbon City Pilot Policy Promote the Formation of Green Lifestyle among Urban Residents? *China Population*, *Resources and Environment*. 2021;31(12), 93–103. Chinese.

[28] Walter Judith Ugelow∙ Environ mental Policies in Developing Countries. *Ambio*. 1979;8(23).

[29] Jing G. Low-carbon City Pilot Policy and Location Choice of Foreign Direct Investment. *East China Economic Management*. 2021;35(12), 43–51. Chinese.

[30] BAI Y SONG S, JIAO J, etal. the impacts of government R&D subsidies on green innovation:evidence from Chinese energy-intensive firms.*J*.*Journal of Cleaner Production*. 2019;233(1):819–829.

[31] Wei M., & Li S. Measurement Research on the Level of High-Quality Economic Development in China in the New Era. *Journal of Quantitative & Technological Economics*. 2018;35(11), 3–20. Chinese.

[32] Sun Y., Zhang P., & Zhang Y. Spatial Pattern and Influencing Factors of Coupled and Coordinated Development of Innovation and Entrepreneurship. *Inquiry into Economic Issues*. 2022;(04), 37–54. Chinese. doi: 10.11821/dlxb201402005

[33] Heckman J J, Ichimura H, Todd P E. Matching as an Econometric Evaluation Estimator: Evidence from Evaluating a Job Training Programme.*J*.*Review of Economic Studies*. 1997;64:605–654.

[34] Heckman J J, Ichimura H, Todd P.Matching as an Econometric Evaluation Estimator. *Review of Economic Studies*. 1998;65:261–294. doi: 10.2307/2971733

[35] Chen S., & Chen D. Haze Pollution, Government Governance, and High-Quality Economic Development. *Economic Research Journal*. 2018;53(02), 20–34. Chinese.

[36] She S., Wang Q., & Zhang A. Technological Innovation, Industrial Structure, and Urban Green Total Factor Productivity: Testing the Impact Channels Based on National Low-Carbon City Pilots. *Research on Economics and Management*. 2020;41(08), 44–61. Chinese.

[37] Wang L., Sun Y., & Xu D. Coupling Coordination and Interactive Response between Green Technology Innovation and High-Quality Green Development. *Journal of Technology Economics*. 2023;42(05), 1–15. Chinese.

[38] Zhu P., Zhang Z., & Jiang G. FDI and Environmental Regulation: An Empirical Study Based on the Perspective of Local Decentralization. *Economic Research Journal*. 2011;46(06), 133–145. Chinese.

