# Supplementary material for: How can low-carbon help high-quality urban development?—Empirical evidence from low-carbon city pilot policies
Source: PLoS One. 2024 May 6;19(5):e0302683. doi: 10.1371/journal.pone.0302683 (PMC11073676; doi:10.1371/journal.pone.0302683)
Supplement: S1 Table — (DOCX) [file pone.0302683.s002.docx]

| **Time** | **Batch** | **City (district, county)** |
| --- | --- | --- |
| July 2010 | The first batch of low-carbon pilot projects | Guangdong Province, Liaoning Province, Hubei Province, Shaanxi Province, Yunnan Province, Tianjin City, Chongqing City, Shenzhen City, Xiamen City, Hangzhou City, Nanchang City, Guiyang City, Baoding City |
| November 2012 | The second batch of low-carbon pilots projects | Beijing, Shanghai, Hainan Province, Shijiazhuang, Qinhuangdao, Jincheng, Hulunbuir, Jilin, Daxing'anling, Suzhou, Huai'an, Zhenjiang, Ningbo, Wenzhou, Chizhou, Nanping, Jingdezhen, Ganzhou, Qingdao, Jiyuan, Wuhan, Guangzhou, Guilin, Guangyuan, Zunyi, Kunming, Yan'an, Jinchang, Urumqi |
| January 2017 | The third batch of low-carbon Pilots projects | Wuhai City, Shenyang City, Dalian City, Chaoyang City, Xunke County, Nanjing City, Changzhou City, Jiaxing City, Jinhua City, Quzhou City, Hefei City, Huaibei City, Huangshan City, Lu'an City, Xuancheng City, Sanming City, Gongqing City, Ji'an City, Fuzhou City, Jinan City, Yantai City, Weifang City, Changyang Tujia Autonomous County, Changsha City, Zhuzhou City, Xiangtan City, Chenzhou City, Zhongshan City, Liuzhou City, Sanya City, Qiongzhong Li and Miao Autonomous County, Chengdu City, Yuxi City, Pu'er City Simao District, Lhasa City, Ankang City, Lanzhou City, Dunhuang City, Xining City, Yinchuan City, Wuzhong City, Changji City, Yining City, Hotan City, First Division Aral City |
