# Supplementary material for: How can low-carbon help high-quality urban development?—Empirical evidence from low-carbon city pilot policies
Source: PLoS One. 2024 May 6;19(5):e0302683. doi: 10.1371/journal.pone.0302683 (PMC11073676; doi:10.1371/journal.pone.0302683)
Supplement: S2 Table — (DOCX) [file pone.0302683.s003.docx]

| **Time** | **City** | **Policy** |
| --- | --- | --- |
| July 2010 | Guiyang City | Guiyang Low Carbon Development Action Plan (Outline) (2010~2020) |
| October 2010 | Baoding City | Baoding Municipal Party Committee and Municipal Government Guiding Opinions on Building a Low-Carbon City |
| December 2011 | Hangzhou City | Hangzhou's "Twelfth Five-Year Plan" low-carbon urban development plan |
| May 2012 | Shenzhen City | Shenzhen Low Carbon Development Medium and Long-term Plan (2011~2020) |
| April 2013 | Shijiazhuang City | Notice on the key points of Shijiazhuang's "Twelfth Five-Year Plan" low-carbon city pilot work |
| April 2013 | Ningbo City | Implementation plan of Ningbo low-carbon city pilot work |
| September 2013 | Wuhan City | Implementation plan for Wuhan low-carbon city pilot work |
| February 2014 | Suzhou City | Suzhou low-carbon development plan |
| September 2014 | Qingdao City | Qingdao Low Carbon Development Plan (2014~2020) |
| April 2017 | Hefei City | Hefei low-carbon city pilot construction work plan |
| May 2017 | Xuancheng City | Xuancheng low-carbon city pilot construction implementation plan |
| November 2017 | Jiaxing City | Jiaxing Three-Year Action Plan for Low-carbon City Pilot Construction (2017-2019) |
| June 2017 | Huaibei City | Huaibei City (2017-2020) low-carbon city pilot construction action plan |
| March 2018 | Yinchuan City | Yinchuan Low-carbon City Development Plan (2017-2020) |
| November 2018 | Jinan city | Jinan Low-carbon Development Work Plan (2018-2020) |
| February 2019 | Changsha city | Changsha Low Carbon Development Plan (2018-2025) |
